# Exercise haemodynamics in pulmonary hypertension – a prospective pressure–volume loop study on right ventricular adaptation and prognosis

**DOI:** 10.1002/ejhf.3802

**Published:** 2025-08-07

**Authors:** Bruno R. Thal, Zvonimir A. Rako, Nils C. Kremer, Athiththan Yogeswaran, Patrick Janetzko, Selin Yildiz, Stephan Rosenkranz, Hossein Ardeschir Ghofrani, Werner Seeger, Friedrich Grimminger, Khodr Tello

**Affiliations:** ^1^ Department of Internal Medicine Justus‐Liebig‐University Giessen, Universities of Giessen and Marburg Lung Center (UGMLC), Institute for Lung Health (ILH), Cardio‐Pulmonary Institute (CPI), Member of the German Center for Lung Research (DZL) Giessen Germany; ^2^ Department of Cardiology – Internal Medicine III, Heart Center University Hospital Cologne Cologne Germany; ^3^ Cologne Cardiovascular Research Center (CCRC), University of Cologne Cologne Germany; ^4^ Department of Pneumology Kerckhoff Heart, Rheuma and Thoracic Center Bad Nauheim Germany; ^5^ Department of Medicine Imperial College London London UK

**Keywords:** Pulmonary hypertension, Right ventricle, Conductance catheterization, Contractile reserve, Exercise, Prognosis

## Abstract

**Aims:**

The haemodynamic response to exercise is prognostic in pulmonary hypertension (PH). However, little is known about right ventricular (RV) adaptation in this context. We analysed the patterns and prognostic relevance of RV adaptation to exercise in PH.

**Methods and results:**

We prospectively analysed 46 patients with PH and 19 disease controls with invasive exclusion of PH. All underwent three‐dimensional echocardiography, pressure–volume catheterization, and right heart catheterization at rest and during stepwise exercise on a semi‐supine ergometer. Patients with PH were classified as homeometric if they had increased RV end‐systolic elastance and preserved RV–pulmonary arterial coupling (end‐systolic/arterial elastance) during exercise (18 patients); otherwise, they were classified as heterometric (28 patients). The mean pulmonary arterial pressure/cardiac output (mPAP/CO) slope was similar in the homeometric and heterometric groups (8.8 [6.5–13.1] vs. 8.6 [4.8–18.8] mmHg·min/L), and lower in disease controls (2.1 [1.1–4.0] mmHg/L). Multivariable logistic regression identified systolic pulmonary arterial pressure change during exercise (ΔsPAP) (odds ratio [OR] 0.93, 95% confidence interval [CI] 0.87–0.99; *p* = 0.019) and peak exercise cardiac index (OR 0.42, 95% CI 0.18–0.97; *p* = 0.042) as key differentiators of homeometric/heterometric adaptation. Heterometric adaptation was significantly associated with clinical worsening and all‐cause mortality (log‐rank *p* = 0.0006 and *p* = 0.0246, respectively) and independently predicted clinical worsening (hazard ratio [HR] 6.52, 95% CI 2.16–19.63; *p* = 0.001); the HR for all‐cause mortality was 6.96 (95% CI 0.87–55.66; *p* = 0.067).

**Conclusions:**

Pulmonary hypertension can present with two RV patterns under stress: homeometric with increased contractile reserve and heterometric with poorer outcome. While the mPAP/CO slope does not differentiate the two, ΔsPAP and peak cardiac index offer potential for RV adaptation pattern identification and thus prognostication.

## Introduction

Right‐sided heart failure is the most frequent cause of mortality in pulmonary hypertension (PH), a pulmonary vascular disease with severely impaired survival rates.^1^, ^2^ Early detection of right ventricular (RV) maladaptation and dysfunction is essential for guiding therapeutic decisions, evaluating treatment efficacy, and estimating prognosis.^1^ Disease progression and many associated symptoms hinge on the ability of the right ventricle to adapt to increasing afterload.^3^, ^4^ Given that symptoms commonly appear during activities such as walking, it becomes pivotal to consider haemodynamic responses under exercise, and not only at rest.

Right heart catheterization (RHC) is the gold standard to assess pulmonary haemodynamics, and exercise RHC has gained attention for facilitating early PH detection and offering additional prognostic information.^1^ Under exercise conditions, pulmonary vascular disease is often characterized by a disproportionate increase in mean pulmonary arterial pressure (mPAP) relative to an insufficient rise in cardiac output (CO). The resulting high mPAP/CO slope is used to help define exercise PH and to identify patients at increased risk.^1^, ^5^, ^6^ The definition of exercise PH was reintroduced in the 2022 European Society of Cardiology/European Respiratory Society (ESC/ERS) guidelines on PH,^1^ and confirmed at the recent 7th World Symposium on PH.^7^

Nevertheless, recent work indicates that relying exclusively on the mPAP/CO slope may overlook the critical influence of the right ventricle itself.^8^ Earlier definitions, which focused on the ability of the right ventricle to increase systolic pulmonary arterial pressure (ΔsPAP) during exercise, provided valuable insights and continue to be strongly supported by current data.^9^ Further research has more thoroughly characterized RV contractile reserve by utilizing end‐systolic elastance (Ees), a load‐independent measure of RV contractility derived from pressure–volume (PV) loops.^10^ In healthy individuals, real‐time PV loop analysis shows that the right ventricle has remarkable contractile and lusitropic reserves. Even under exercise, stroke volume (SV) remains stable through adjustment of Ees and relaxation, without excessive dilatation.^11^ Notably, patients with impaired RV contractile reserve have been reported to exhibit considerable RV dilatation during exercise.^10^ Classic concepts of RV physiology distinguish, particularly in the context of chronic stress, between homeometric and heterometric adaptation. Homeometric adaptation is characterized by concentric hypertrophy that increases contractility with nearly unchanged end‐diastolic volume (EDV), thereby maintaining RV–pulmonary arterial (PA) coupling.^12^, ^13^ By contrast, heterometric adaptation is volume‐mediated through the Frank–Starling mechanism; this occurs when the contractile reserve is exhausted and is accompanied by progressive dilatation.^14^, ^15^

In this prospective study, we sought to assess the RV response to exercise comprehensively using gold‐standard PV catheterization in combination with RHC and three‐dimensional (3D) echocardiography. We considered that the observed (homeometric vs. heterometric) adaptations might be clinically relevant and not fully captured by conventional clinical methods.

## Methods

### Study design and patients

**Figure 1:**
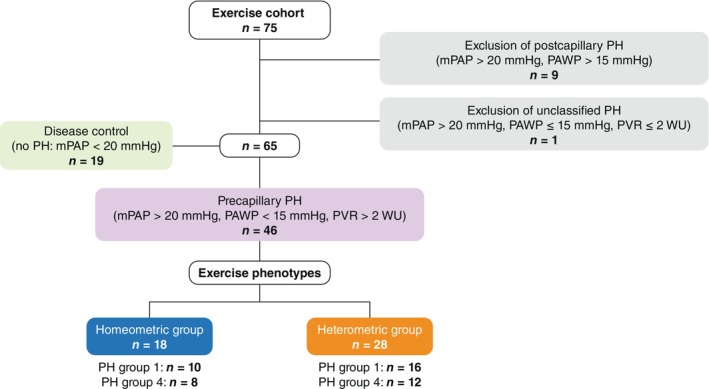
Study flow chart. mPAP, mean pulmonary arterial pressure; PAWP, pulmonary arterial wedge pressure; PH, pulmonary hypertension; PVR, pulmonary vascular resistance.

We prospectively analysed consecutive patients enrolled into the EXERTION study (Exercise Hemodynamic, Right Ventricular Coupling and Echocardiography in Pulmonary Hypertension; ClinicalTrials.gov identifier: NCT04663217) between November 2020 and March 2022. All participating patients gave written informed consent. Diagnoses of pulmonary arterial hypertension (PAH) and chronic thromboembolic PH were assessed by a multidisciplinary board and updated post hoc according to the latest guidelines.^1^, ^16^, ^17^ Patients without PH were initially referred for suspicion of PH and dyspnoea on exertion, but subsequent invasive assessment excluded the presence of pulmonary vascular disease (mPAP ≤20 mmHg and pulmonary vascular resistance [PVR] <2 WU). They formed our ‘disease controls’, as they presented with clinical suspicion of PH but were ultimately found not to meet current haemodynamic criteria for PH. Exercise haemodynamics were not considered for classification of this group; individuals without PH at rest but fulfilling the definition of exercise PH (mPAP/CO slope >3 WU) during exertion remained in the disease control group.^18^ The study conforms with the principles outlined in the Declaration of Helsinki and was approved by the Ethics Committee of the Department of Medicine at the University of Giessen (approval number 117/16). The patients underwent a uniform diagnostic assessment including 3D echocardiography (according to current guidelines^19^) and cardiopulmonary exercise testing if feasible, 1 day before RHC. *Figure* *1* provides an overview of the inclusion and exclusion of patients in this study.

### Right heart catheterization

All patients underwent RHC in accordance with the standards of the local expert centre.^20^ A Swan–Ganz catheter was inserted via the internal jugular vein using an 8F introducer sheath. Pressure parameters including mPAP, right atrial pressure (RAP), pulmonary arterial wedge pressure (PAWP), sPAP, and diastolic pulmonary arterial pressure (dPAP) were continuously monitored. CO was assessed via thermodilution, with averaging of at least three to five measurements.^1^, ^21^ PVR was calculated as (mPAP − PAWP)/CO. Cardiac index was calculated as CO/body surface area (BSA).^22^ BSA was determined using the Du Bois method.^23^ Patients followed a standardized exercise protocol on a semi‐supine ergometer, starting at an initial load of 15–20 W and increasing in increments of 10–20 W every 2 min. The protocol was terminated either upon reaching a symptomatically limited exercise maximum or after 12 min of exercise time.

### Echocardiography

Echocardiography was conducted using a Philips Epiq 7G Elite (Philips Healthcare, Best, The Netherlands) in accordance with current guidelines.^19^ For 3D RV volume parameters, apical four‐ and two‐chamber views were employed at end‐diastole. Specific points on the RV apex and the tricuspid annulus line were identified. The software integrated into the Epiq 7G Elite reconstructed the RV endocardial surface and automatically calculated RV volumes and functions. Manual adjustments were made as needed.^24^

### Pressure–volume catheterization

To generate PV loops, we inserted a 4 F PV catheter (Catalogue No. 41063, CD‐Leycom, Zoetermeer, The Netherlands) via the same 8F sheath employed for RHC. The catheter tip was oriented at the RV apex, guided by transthoracic echocardiography.^25^ PV loops were acquired in real time through the Inca device (CD Leycom, Zoetermeer, The Netherlands). This enabled beat‐to‐beat adjustments and archiving of PV loops for subsequent analyses. 3D echocardiography was employed for volume calibration.^26^ Signal‐averaged PV loops were derived from at least three acceptable beats to reduce respiratory variations. The catheter tip was oriented towards the RV apex under transthoracic echocardiographic guidance, and its position was continuously re‐evaluated throughout the recording, including during training phases and Valsalva manoeuvres, either by continuous imaging or by reviewing the segmental loop morphology. If wall contact was detected during imaging, or if the PV loop showed a secondary pressure peak, an unexplained volume drop during an isovolumetric phase, or a loss of the expected four‐phase morphology, the catheter was carefully rotated, advanced, or retracted under echocardiographic guidance to restore a central, free‐floating position.^25^ Trapezoidal loops in a counterclockwise direction confirmed correct intraventricular placement; clockwise loops or figure‐of‐eights were excluded as signs of misplacement (e.g. in the atrium or outflow tract). Segments outside the right ventricle (e.g. with systolic volume increase) were excluded. Recordings lasted at least 10 s in sinus rhythm and 15 s in atrial fibrillation to ensure consistent cycles. Due to stable intrathoracic pressure, loops at the end of exhalation were preferred, and extrasystoles were avoided by slightly withdrawing the catheter if mechanical stimulation was suspected. During dynamic exercise tests, the catheter was fixed to prevent displacement.^25^, ^27^

Diastolic stiffness, measured as end‐diastolic elastance (Eed), was calculated via exponential fitting of the end‐diastolic PV relationship, as previously described by our group.^28^ RV end‐systolic elastance (Ees) was obtained through multi‐beat PV loop analysis following the execution of a Valsalva manoeuvre at rest and during exercise. The Valsalva manoeuvre resulted in a linear regression line of left‐shifted end‐systolic PV points, representing the end‐systolic PV relationship (ESPVR). Ees was calculated as the slope of the ESPVR.^10^, ^29^ Arterial elastance (Ea) was determined by dividing end‐systolic pressure (ESP) by RV SV.^30^ The RV Ees/Ea ratio is indicative of RV‐PA coupling.^31^ Systolic ejection time was derived from ensemble averaged PV loops as the interval from the R wave to the peak of instantaneous elastance and was corrected for heart rate using the Fridericia formula, following the approach previously described by Reil *et al*.^32^, ^33^ (online supplementary *Figure* *S1*).

Loops were generally excluded when data integrity appeared uncertain, that is when they (i) exhibited pronounced noise or signal interference, (ii) showed pressure or volume excursions outside physiological ranges, (iii) contained suspected artefact‐related pressure spikes, (iv) displayed abrupt volume changes without a clear physiological basis, (v) coincided with arrhythmic beats, (vi) were captured outside end‐expiration, or (vii) demonstrated segmental loop patterns suggestive of catheter malposition.^25^

### Group classification

To analyse how patients respond to increasing afterload during exercise, we focused on two key parameters. First, we measured the change in Ees from rest to exercise (ΔEes) to quantify RV contractile reserve. Second, we evaluated the change in the Ees/Ea ratio (ΔEes/Ea), to assess the ability of the right ventricle to maintain RV‐PA coupling under rising afterload. Although these measurements are continuous in nature, we have selected thresholds to define two primary forms of RV adaptation for pragmatic analysis, without implying strict pathophysiological subtypes. Based on these parameters, patients with PH were divided into two groups. The homeometric group was defined by a positive ΔEes (>0 mmHg/ml) coupled with an unchanged or increased Ees/Ea ratio, indicating preserved RV‐PA coupling. All other patients with PH were assigned to the heterometric group.

### Risk stratification and outcomes

We used two risk stratification methods to evaluate the risk profile of the studied cohort. The first method is the REVEAL Lite 2 score, which offers clinicians an approach to calculate risk in clinical settings.^34^ The second method is based on guidelines provided by the ESC/ERS.^35^

To assess prognostic significance, we performed two separate analyses in our cohort. First, we analysed clinical worsening, defined as the time to the first occurrence of any of the following events: (1) a reduction of at least 15% in the 6‐min walk distance; (2) deterioration in New York Heart Association (NYHA) functional class; (3) hospitalization due to clinical worsening; (4) adjustments in PH medication; (5) requirement for diuretics; (6) lung transplantation; or (7) death. Second, we performed a survival analysis, in which death from any cause was recorded from the time of RHC. We closely monitored the cohort for these events until 5 February 2025.

### Statistical analysis

We assessed distribution using the Shapiro–Wilk test. Data that followed a normal distribution are presented as mean ± standard deviation, and group comparisons of these variables were performed using paired *t*‐tests. For non‐normally distributed parameters, data are presented as median (first quartile, third quartile), and the Mann–Whitney U test was used for group comparisons. Categorical variables were compared with the chi‐square test.

In order to analyse both group differences (homeometric vs. heterometric) and the effect of movement (rest vs. peak performance) within a single framework, we additionally performed a factorial ANOVA. Specifically, we treated rest versus peak as the factor within patients (within condition) and the group as the factor between patients, generating *p*‐values for the main effect of the group, the main effect of the condition, and the interaction term (group × condition). To identify predictors distinguishing the homeometric and heterometric groups, we conducted univariate logistic regression analyses (adjusted for age, sex, and haemoglobin level) and selected variables with *p* < 0.10 for multivariate analysis. Variance inflation factor (VIF) checks for collinearity and backward elimination were then performed to arrive at a final multivariable model. Model performance was evaluated via receiver operating characteristic (ROC) analysis.

For time‐to‐event endpoints, we utilized Cox proportional hazards regression with the same scheme, covariate sequence, and adjustments used in the logistic regression. After initial univariate analyses (with VIF‐based screening), relevant predictors entered a backward‐elimination multivariable Cox model. We reported hazard ratios (HR) with 95% confidence intervals (CI) and verified the proportional hazards assumption. Kaplan–Meier analyses and log‐rank tests were also performed to compare overall and event‐free survival between groups.

All analyses were carried out using SPSS version 29.0 (IBM, Armonk, NY, USA) and Python 3.12.1 (Python Software Foundation, 2023), with *p* < 0.05 considered statistically significant.

## Results

### Study population

A total of 65 patients were enrolled in this study, including 26 with group 1 PH and 20 with group 4 PH (*Table* *1*). In 19 cases, PH was definitively ruled out by invasive testing; these patients formed the disease control group. Six of these met criteria for exercise PH but were retained in the disease control group. The median age of the overall cohort was 67 (57–74) years, and the majority of the participants were female (66%). Functionally, most were categorized in NYHA class III (58%) (*Table* *1*).

**Table 1:** Patient characteristics

Patient characteristics	Total	Disease control	Homeometric group	Heterometric group	*p*‐value^a^
Patients, *n* (%)	65 (100)	19 (29)	18 (28)	28 (43)	
Male/female	22/43	6/13	5/13	11/17	0.629^b^
Age, years	67.0 (57.0–74.0)	60.0 (44.5–71.5)	62.5 (57.8–73.2)	69.5 (60.8–77.8)	0.149^c^
BMI, kg/m^2^	27.7 (23.6–32.2)	28.4 (25.2–31.5)	29.6 (24.1–32.1)	26.0 (23.3–33.3)	0.669^c^
BNP, pg/ml	72.0 (20.0–169.0)	25.0 (16.0–47.5)	69.0 (17.8–152.2)	133.5 (65.2–264.8)	0.126^c^
Haemoglobin, g/L	140.0 (131.0–147.0)	141.0 (136.0–149.0)	138.0 (131.0–145.8)	137.0 (131.8–147.0)	0.95^c^
eGFR, ml/min/1.73 m^2^	79.8 ± 27.8	93.3 ± 25.4	76.5 ± 26.5	72.7 ± 27.9	0.65^d^
Borg dyspnoea score (peak)	7.0 (5.0–9.0)	6.0 (5.0–7.0)	7.0 (5.0–8.0)	8.5 (7.0–9.2)	0.041^c^
Performance (semi‐supine ergometer), W/kg	0.9 (0.5–1.5)	1.3 (0.8–1.9)	1.0 (0.7–1.5)	0.6 (0.4–1.0)	0.052^c^
Risk scores					
REVEAL Lite 2	6.0 ± 2.1	4.5 ± 1.6	5.9 ± 2.0	7.1 ± 1.9	0.051^d^
ESC/ERS 2015 guidelines	1.6 (1.4–2)	1.4 (1.2–1.5)	1.6 (1.4–1.8)	2.0 (1.7–2.3)	<0.001^c^
Non‐PH, *n* (%)	19 (29)	19 (100)			
PH subtype, *n* (%)					
PH group 1	26 (40)		10 (56)	16 (57)	
PH group 4	20 (31)		8 (44)	12 (43)	
NYHA class, *n* (%)					
I	5 (8)	2 (11)	3 (17)		
II	18 (28)	9 (47)	4 (22)	5 (18)	
III	38 (58)	7 (37)	11 (61)	20 (71)	
IV	4 (6)	1 (5)		3 (11)	

Values represent mean ± standard deviation or median (first quartile–third quartile), unless otherwise specified.

BMI, body mass index; BNP, brain natriuretic peptide; eGFR, estimated glomerular filtration rate; ESC/ERS, European Society of Cardiology/European Respiratory Society; NYHA, New York Heart Association; PH, pulmonary hypertension.

^a^ Homeometric versus heterometric group.

^b^ Chi‐square test.

^c^ Mann–Whitney U test.

^d^ *t*‐test.

Based on predefined criteria, 18 patients with PH were assigned as homeometric and 28 as heterometric. There were no significant differences in sex distribution or age between the two groups. *Table* *1* compares further patient characteristics between the homeometric and heterometric groups.

The median follow‐up time was 41.50 (36.50–43.75) months, during which 27 patients had documented clinical worsening. Hospitalization was the most frequent cause of clinical worsening (7 patients, 26%), followed by escalation in PH medication and a reduction in the 6‐min walk distance (each in 6 patients, 22%). During the observation period, one patient in the homeometric group and 10 in the heterometric group died.

### Resting conditions: comparable pressures but distinct cardiac function in homeometric versus heterometric groups

Resting RHC measures stratified by homeometric versus heterometric function (*Table* *2*, *Graphical Abstract*) revealed significantly higher CO and cardiac index in the homeometric group, despite comparable pressure (mPAP) and afterload (PVR) parameters. PAWP was significantly higher in the heterometric group, while remaining in the range defining pre‐capillary PH. No differences emerged in left ventricular parameters (online supplementary *Table* *S1*). Nearly all of the patients with PH exhibited some degree of tricuspid regurgitation at rest (grade 1–3), with no difference between the homeometric and heterometric groups (*p* = 0.702; online supplementary *Figure* *S2*).

**Table 2:** Resting and peak haemodynamic data with factorial ANOVA results (right heart catheterization and echocardiography)

Variables	Rest			*p* ^a^ Homeometric vs. heterometric	Peak			*p* ^a^ Homeometric vs. heterometric	*p* ^a^, ^e^ between‐ groups	*p* ^a^, ^f^ within‐ condition	*p* ^a^, ^g^ group x condition
	Disease control	Homeometric group	Heterometric group		Disease control	Homeometric group	Heterometric group				
RHC											
Heart rate, bpm	69.6 ± 14.1	73.2 ± 15.6	70.0 ± 12.0	0.437^b^	109.8 ± 25.5	102.8 ± 23.7	94.8 ± 20.3	0.225^b^	0.229	<0.001	0.409
mRAP, mmHg	5.4 ± 2.8	5.5 ± 3.0	8.5 ± 4.3	0.014^b^	6.6 ± 3.4	11.8 ± 4.7	15.6 ± 7.6	0.067^b^	0.027	<0.001	0.599
mPAP, mmHg	16.5 ± 2.7	38.9 ± 11.9	40.2 ± 12.1	0.729^b^	27.1 ± 7.0	61.9 ± 18.1	58.1 ± 16.1	0.462^b^	0.767	<0.001	0.078
sPAP, mmHg	26.9 ± 4.4	66.2 ± 23.1	65.9 ± 19.0	0.970	42.8 ± 11.4	104.4 ± 30.4	93.8 ± 24.8	0.199^b^	0.435	<0.001	0.023
PAWP, mmHg	7.8 ± 2.5	8.3 ± 3.5	11.0 ± 3.2	0.011^b^	11.5 ± 6.0	12.4 ± 4.7	15.1 ± 6.5	0.139^b^	0.041	<0.001	0.991
CO, L/min	5.3 (4.3–5.9)	5.2 (4.5–5.8)	4.0 (3.6–4.7)	0.004^c^	9.9 ± 3.6	7.9 ± 2.0	6.4 ± 2.2	0.019^b^	0.015	<0.001	0.104
Cardiac index, L/min/m^2^	3.0 (2.5–3.3)	2.7 (2.5–3.1)	2.5 (2.2–2.8)	0.041^c^	5.0 ± 1.8	4.1 ± 0.8	3.3 ± 1.0	0.008^b^	0.006	<0.001	0.288
PVR, dyn·s/cm^5^	136.1 (107.8–150.5)	435.1 (300.3–737.4)	519.3 (365.7–825.4)	0.362^c^	124.1 (96.2–143.6)	483.2 (404.3–704.2)	484.2 (333.2–805.5)	0.661^c^	0.382	0.032	0.813
RHC – response to exercise											
mPAP/CO slope, mmHg/L/min					2.1 (1.1–4.0)	8.8 (6.5–13.1)	8.6 (4.8–18.8)	0.884^c^			
PAWP/CO slope, mmHg/L/min					0.5 (0.0–1.2)	1.4 (0.3–2.3)	0.9 (0.0–3.4)	0.982^c^			
TPG/CO slope					1.4 (0.9–2.4)	7.3 (5.6–9.8)	6.1 (3.3–16.7)	0.566^c^			
ΔsPAP, mmHg					15.9 ± 9.9	38.3 ± 17.5	27.8 ± 12.6	0.023^b^			
Echocardiography											
RV FWLS, %	−27.7 ± 5.3	−22.7 ± 4.4	−19.5 ± 5.0	0.033^b^	−28.6 (−34.3 to −26.8) (*n* = 5)^d^	−25.1 (−26.9 to −19.3) (*n* = 2)^d^	−20.9 (−23.4 to −13.4) (*n* = 4)^d^	0.029^c^	0.009	0.606	0.494
3D RV EF, %	50.1 ± 5.6	44.1 ± 8.6	39.9 ± 10.0	0.157^b^	48.6 ± 7.1 (*n* = 2)^d^	42.6 ± 10.4 (*n* = 3)^d^	36.2 ± 9.8 (*n* = 3)^d^	0.061^b^	0.119	0.002	0.126

Values represent mean ± standard deviation or median (first quartile–third quartile), unless otherwise specified. Δ (change during exercise) indicates the difference between rest and peak exercise measurements for the respective parameter. Further exercise RHC parameters are shown in online supplementary *Table* *S3*.

3D, three‐dimensional; CO, cardiac output (assessed by thermodilution); EF, ejection fraction; FWLS, free wall longitudinal strain; mPAP, mean pulmonary arterial pressure; mRAP, mean right atrial pressure; PAWP, pulmonary arterial wedge pressure; PVR, pulmonary vascular resistance; RHC, right heart catheterization; RV, right ventricular; sPAP, systolic pulmonary arterial pressure; TPG, transpulmonary gradient.

^a^ Homeometric versus heterometric group.

^b^ *t*‐test.

^c^ Mann–Whitney U test.

^d^ Number of participants with missing data.

^e^ Between‐groups *p*‐value from factorial ANOVA (overall effect of homeometric vs. heterometric).

^f^ Within‐condition *p*‐value from factorial ANOVA (effect of rest vs. peak across both groups).

^g^ Group × condition *p*‐value from factorial ANOVA (interaction indicating whether the two groups differ in their response to exercise).

Echocardiographically, the homeometric group showed better RV free wall longitudinal strain (FWLS) than the heterometric group, whereas 3D RV ejection fraction (EF) remained similar (*Table* *2*). PV data indicated no intergroup differences in end‐systolic volume (ESV), EDV, Ees/Ea ratio, Ees, Eed, or Ea; however, the heterometric group demonstrated a significantly higher end‐diastolic pressure at rest (*Table* *3*).

**Table 3:** Resting and peak haemodynamic data with factorial ANOVA results (pressure–volume catheterization)

Variables	Rest			*p* ^a^ Homeometric vs. Heterometric	Peak			*p* ^a^ Homeometric vs. Heterometric	*p* ^a^, ^d^ between‐ groups	*p* ^a^, ^e^ within‐ condition	*p* ^a^, ^f^ group × condition
	Disease control	Homeometric group	Heterometric group		Disease control	Homeometric group	Heterometric group				
PV catheterization											
ESP, mmHg	20.6 ± 7.5	56.7 ± 22.7	58.4 ± 20.9	0.804^b^	35.3 ± 12.1	91.2 ± 24.3	81.8 ± 19.7	0.154^b^	0.537	<0.001	0.003
EDP, mmHg	3.0 (2.0–6.5)	5.5 (3.2–7.5)	9.0 (5.8–14.0)	0.004^c^	6.5 ± 6.7	13.9 ± 7.3	17.7 ± 9.3	0.158^b^	0.029	<0.001	0.687
ESV, ml	49.5 ± 18.5	83.1 ± 28.8	88.4 ± 35.2	0.600^b^	54.8 ± 18.8	83.3 ± 32.6	109.4 ± 42.4	0.032^b^	0.144	<0.001	<0.001
EDV, ml	105.0 ± 29.4	143.9 ± 41.8	143.2 ± 48.3	0.959^b^	105.6 ± 27.7	147.8 ± 41.7	164.3 ± 46.3	0.228^b^	0.556	<0.001	0.010
SV, ml	69.6 ± 16.5	78.8 ± 25.3	80.0 ± 20.6	0.869^b^	77.5 ± 29.4	99.9 ± 36.2	76.1 ± 25.7	0.012^b^	0.109	0.143	0.004
Eed, mmHg/ml	0.1 (0.1–0.2)	0.2 (0.1–0.3)	0.2 (0.1–0.3)	0.339^c^	0.2 (0.1–0.3)	0.3 (0.2–0.3)	0.5 (0.3–0.6)	0.006^c^	0.008	<0.001	0.025
Ees, mmHg/ml	0.4 (0.3–0.7)	0.7 (0.6–0.9)	0.8 (0.6–1.0)	0.744^c^	0.8 ± 0.4	1.3 ± 0.4	0.9 ± 0.3	<0.001^b^	0.077	<0.001	<0.001
Ea, mmHg/ml	0.3 (0.2–0.4)	0.7 (0.4–1.1)	0.6 (0.5–0.9)	0.744^c^	0.5 (0.3–0.7)	1.0 (0.7–1.2)	1.0 (0.9–1.3)	0.242^c^	0.501	<0.001	0.073
Ees/Ea	1.6 ± 0.5	1.1 ± 0.3	1.1 ± 0.3	0.791^b^	1.7 ± 0.6	1.4 ± 0.4	0.9 ± 0.4	<0.001^b^	0.020	0.193	<0.001
V0, ml	2.0 ± 13.2	6.8 ± 23.7	15.0 ± 26.9	0.300^b^							
PV catheterization – response to exercise											
ΔESP, mmHg					14.6 ± 8.3	34.5 ± 11.2	23.4 ± 11.8				
ΔEDP, mmHg					2.7 ± 4.3	9.2 ± 6.9	8.3 ± 6.9				
ΔESV, ml					0.0 (−7.5 to 16.0)	2.0 (−2.5 to 5.5)	21.0 (6.8–29.5)				
ΔEDV, ml					7.0 (−15.0, 17.0)	4.0 (−7.0 to 17.0)	26.5 (2.8– 38.8)				
ΔSV, ml					7.9 ± 24.6	21.1 ± 23.1	−3.8 ± 29.2				
ΔEed, mmHg/ml					0.1 ± 0.1	0.1 ± 0.2	0.2 ± 0.2				
ΔEes, mmHg/ml					0.3 ± 0.4	0.5 ± 0.3	0.1 ± 0.3				
ΔEa, mmHg/ml					0.1 (0.1–0.3)	0.2 (0.1–0.3)	0.3 (0.2–0.6)				
ΔEes/Ea					0.1 ± 0.5	0.3 ± 0.2	−0.3 ± 0.3				

Values represent mean ± standard deviation or median (first quartile–third quartile), unless otherwise specified. Δ (change during exercise) indicates the difference between rest and peak exercise measurements for the respective parameter. Systolic ejection time is shown in online supplementary *Table* *S4*.

Ea, arterial elastance; EDP, end‐diastolic pressure; EDV, end‐diastolic volume; Eed, end‐diastolic elastance; Ees, end‐systolic elastance; ESP, end‐systolic pressure; ESV, end‐systolic volume; PV, pressure–volume; RV, right ventricular; SV, stroke volume; V0, x‐intercept of the end‐systolic pressure–volume relationship.

^a^ Homeometric versus heterometric group.

^b^ *t*‐test.

^c^ Mann–Whitney U test.

^d^ Between‐groups *p*‐value from factorial ANOVA (overall effect of homeometric vs. heterometric).

^e^ Within‐condition *p*‐value from factorial ANOVA (effect of rest vs. peak across both groups).

^f^ Group × condition *p*‐value from factorial ANOVA (interaction indicating whether the two groups differ in their response to exercise).

### Exercise conditions: varied responses in homeometric versus non‐homeometric groups

Changes in haemodynamics and RV function from rest to peak exercise are shown in *Tables* *2*, *3*, online supplementary *Table* *S2* and in the *Graphical Abstract*. Notably, RV EF significantly decreased and RV volumes significantly increased during exercise in the heterometric group but not in the homeometric or disease control groups.

Under exercise conditions, the homeometric group demonstrated significantly higher CO and cardiac index than the heterometric group, despite similar mPAP, PVR, and PAWP (*Table* *2*). Slope analyses (mPAP/CO, PAWP/CO, and transpulmonary gradient [TPG]/CO) revealed no significant intergroup differences. Echocardiographic findings showed worse RV FWLS and a trend towards lower 3D RV EF (*p* = 0.061) in the heterometric group compared with the homeometric group, although changes in these values from rest to exercise did not show any significant differences between the RV adaptation groups (*p* = 0.494 and *p* = 0.126, respectively).

By definition, the heterometric group had significantly lower Ees/Ea and ΔEes/Ea during exercise than the homeometric group, driven by significantly lower Ees and ΔEes (*Table* *3*). However, Ea did not differ significantly between the two groups, although the difference in ΔEa between the RV adaptation groups almost reached significance in the ANOVA factorial analysis (*p* = 0.073). During exercise, the heterometric group also exhibited a marked rise in diastolic stiffness (ΔEed), larger increases in ESV and EDV, and a reduction in ΔSV compared with the homeometric group. Meanwhile, the homeometric group showed a more pronounced increase in ESP and sPAP. EDP changes were similar between the two groups (*Table* *3*).

### Functional capacity, symptom burden, and risk profile

Brain natriuretic peptide levels were comparable between groups (*Table* *1*). Nevertheless, distinct differences emerged in functional and risk parameters. The homeometric group exhibited a lower peak Borg dyspnoea score and tended to achieve higher performance on the semi‐supine ergometer than the heterometric group. By contrast, the heterometric group demonstrated significantly higher ESC/ERS guideline‐based risk scores and a trend towards higher REVEAL Lite 2 scores compared with the homeometric group (*Table* *1*). With both scoring systems, the distribution of risk levels (low, intermediate, and high) differed significantly across the study groups, with the worst risk distribution in the heterometric group (*p* < 0.001; online supplementary *Figure* *S3*).

### Logistic regression‐based classification prediction: higher ΔsPAP and cardiac index indicate homeometric function

**Figure 2:**
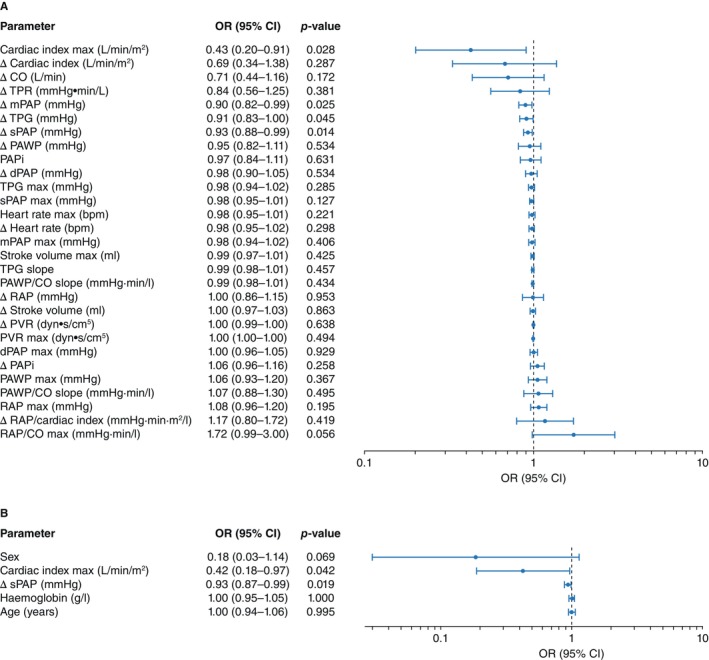
Logistic regression analysis of potential haemodynamic predictors of right ventricular adaptation. (*A*) Univariate logistic regression (adjusted for age, sex, and haemoglobin level) was performed for each candidate haemodynamic parameter, including peak exercise and response‐to‐exercise parameters. Parameters with *p* < 0.10 were retained and assessed for multicollinearity using variance inflation factor. (*B*) Backward elimination was then performed to arrive at a final multivariable model. An odds ratio (OR) >1 indicates higher odds of a heterometric response, while an OR <1 suggests a homeometric response. CI, confidence interval; CO, cardiac output; dPAP, diastolic pulmonary arterial pressure; mPAP, mean pulmonary arterial pressure; PAPi, pulmonary arterial pulsatility index; PAWP, pulmonary arterial wedge pressure; PVR, pulmonary vascular resistance; RAP, right atrial pressure; sPAP, systolic pulmonary arterial pressure; TPG, transpulmonary gradient; TPR, total pulmonary resistance.

To investigate whether haemodynamics at peak exercise or haemodynamic changes in response to exercise could differentiate homeometric from heterometric adaptation, we performed logistic regression analyses (*Figure* *2*). Lower ΔsPAP (odds ratio [OR] 0.93, *p* = 0.019) and lower cardiac index at peak exercise (OR 0.42, *p* = 0.042) remained significant predictors of heterometric status in the final model, indicating that higher ΔsPAP and higher cardiac index at peak exercise favour the homeometric RV adaptation. The final model distinguished the two RV adaptation patterns with an area under the ROC curve (AUC) of 0.82, an optimal threshold of 0.75, a sensitivity of 0.64, and a specificity of 0.89 (online supplementary *Figure* *S4*).

### Logistic regression‐based classification with non‐invasive surrogate markers and functional class

To investigate whether echocardiographic measurements could differentiate homeometric from heterometric adaptation, we performed logistic regression analyses on a broad range of echocardiographic parameters. Lower peak 3D SV to ESV ratio (OR 0.10, *p* = 0.072) was associated with heterometric status, indicating that higher values favour the homeometric response. The final model distinguished the two adaptation patterns with an AUC in ROC analysis of 0.71, an optimal threshold of 0.53, a sensitivity of 0.92, and a specificity of 0.53. When NYHA functional class was introduced into the same selection procedure it emerged as the sole predictor (OR 4.29 [95% CI 1.12–16.40], *p* = 0.033) but lowered discrimination to an AUC of 0.68 (optimal threshold 0.69, sensitivity 0.88, and specificity 0.40).

### Follow‐up: Cox proportional hazards analysis confirms adaptation‐based risk stratification

**Figure 3:**
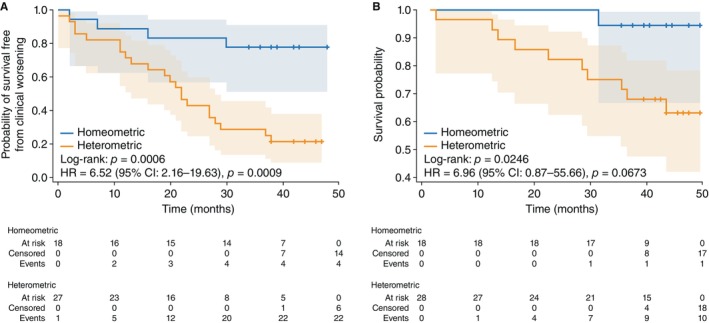
Kaplan–Meier analyses of (*A*) clinical worsening and (*B*) survival in homeometric versus heterometric groups. Cox hazard ratios (HR) were adjusted for age, sex, and haemoglobin level. CI, confidence interval.

To determine whether the defined classification remains a robust predictor of clinical worsening, we performed Cox proportional hazards analyses. In the final model, this classification retained significance (HR 6.52 [95% CI 2.16–19.63], *p* = 0.0009), indicating that the heterometric group has a higher risk for clinical worsening than the homeometric group. These results were confirmed by Kaplan–Meier analysis (log‐rank *p* = 0.0006; *Figure* *3*).

In a separate Cox model evaluating overall survival, the HR was 6.96 (95% CI 0.87–55.66; *p* = 0.0673), slightly above the significance threshold, while the corresponding Kaplan–Meier analysis showed significantly higher mortality in the heterometric group compared with the homeometric group (log‐rank *p* = 0.0246; *Figure* *3*).

## Discussion

Our results highlight three key points that are critical to understanding the RV response to exercise and its prognostic significance. First, based on gold‐standard PV analysis, we identified two clearly distinguishable patterns of RV adaptation under exercise conditions. Second, while the mPAP/CO slope provides additional value in differentiating individuals with exercise‐induced PH from those without resting PH, our study in patients with advanced PH demonstrates that the capacity of the right ventricle to increase pressure under stress, defined as ΔsPAP, and peak cardiac index are important predictors of each patient's specific RV adaptation. Third, after an overall median follow‐up of about 41 months, our RV classification showed that patients with a heterometric RV response to exercise are at a significantly higher risk of clinical deterioration and show a marked tendency towards increased mortality compared with patients who have a homeometric RV response.

In the clinical and scientific discussion of PH, the main focus is on resting haemodynamic parameters or load‐dependent parameters such as the mPAP/CO slope.^1^, ^5^ Although it is now well established that the dynamics of the pressure‐flow relationship during exercise are prognostically relevant, particularly in mild PH, there is still a significant gap in the targeted analysis of RV adaptation and ability to respond to exercise, as well as their clinical implications.^5^, ^6^ Our study addresses this research gap by examining RV behaviour during stepwise exercise in a semi‐supine position and, in particular, by assessing whether the classic mPAP/CO slope, which has already proven valuable in identifying exercise‐induced PH in patients with excluded resting PH, is sufficient to characterize the heterometric and homeometric RV adaptation types in advanced PH, or whether the characterization and prognostication can be further refined by additional parameters that assess the RV response to stress in an unbiased manner.^6^

Our data show two clearly distinct RV adaptation patterns during exercise. The homeometric group has a higher contractile reserve than the heterometric group by definition, with the capacity to increase Ees during exercise while maintaining largely stable RV volumes and preserving the Ees/Ea ratio. This group shows significantly higher CO and peak cardiac index and is directly associated with a lower incidence of clinical events compared with the heterometric group. By contrast, the heterometric group shows only limited or no increase in RV contractility during exercise and, on average, marked RV dilatation with increases in ESV and EDV. In response to exercise, this group demonstrates a reduction in the Ees/Ea ratio, indicative of RV‐PA uncoupling. Increased Eed also points to enhanced stiffness due to heterometric adaptation. Clinically, these patients exhibit reduced exercise capacity, increased dyspnoea, and a markedly higher rate of clinical deterioration than the homeometric group, with a clear trend towards poorer survival (an effect we could only approximate owing to the relatively short observation period).

The oft‐cited distinction between homeometric and heterometric mechanisms is clearly confirmed in our exercise data.^14^ In the case of homeometric adaptation, the right ventricle can compensate for the increasing afterload pressure by increasing its contractility (Ees) and avoiding dilatation.^3^, ^10^, ^36^ Human studies of hypertrophic obstructive cardiomyopathy describe a chronic Anrep response, characterized by increased Ea and Ees alongside a prolonged systolic ejection time, forming a triad indicative of homeometric adaptation.^32^, ^37^ In our PH cohort, systolic ejection time was likewise modestly prolonged in the homeometric group compared with the disease control group (online supplementary *Table* *S4*), thereby completing the mentioned triad. However, its duration remained stable from rest to exercise, suggesting that SV is maintained predominantly through enhanced contractility rather than progressive systolic lengthening. By contrast, the heterometric group shows a volume‐related adaptation pathway that initially maintains SV via the Frank–Starling mechanism, but in the long term can lead to increasing filling pressures and RV‐PA uncoupling.^36^, ^38^ This observation is consistent with Hsu *et al*.,^29^ who found limited contractile reserve and RV dilatation in systemic sclerosis‐associated PAH using multi‐beat PV loops.

Recently published work has demonstrated that the mPAP/CO slope is a meaningful prognostic variable in early or mild stages of PH, specifically in patients with normal or only mildly elevated resting mPAP in whom PH at rest has been excluded.^5^, ^6^ In our group with advanced PH, however, this parameter is less meaningful because some of these patients already have chronically elevated resting pressure with PVR above 3 WU.^39^ Our results suggest that contractility‐sensitive parameters such as ΔsPAP and peak cardiac index are better able to distinguish between RV adaptation types and thus have predictive value in this group; our findings are also consistent with studies that have emphasized these parameters in terms of prognosis.^9^, ^40^ Spruijt *et al*.^41^ hypothesized that the lack of increase in RV contractility with increasing afterload explains the limited physical capacity in PAH. However, their findings are based solely on pressure‐based Ees estimates without simultaneous PV curves, which limits interpretation but does not invalidate the core finding. Studies on dynamic RV‐PA uncoupling also support the notion that pressure‐dominated analysis is no longer sufficient in later stages of PH. These studies indicate that with increasing afterload, the RV contractile reserve is often no longer sufficient, which consequently leads to a reduction in SV.^39^ To accurately assess load‐independent RV contractility, complete volume and pressure measurements are essential, as V0 (the x‐intercept of the ESPVR) in patients with PAH is not negligible owing to RV dilatation.^42^

Another important aspect is the type of exercise. We used a semi‐supine exercise protocol which allowed us to perform simultaneous invasive pressure and volume measurements in a stable body position. Previous studies suggest that in an upright position, an even higher afterload often results, which might have further accentuated differences between homeometric (contractile) and heterometric (dilating) RV responses.^43^ Our results are therefore likely to represent a conservative estimate of the actual differences in adaptation.

Furthermore, a recent study by Ireland *et al*.^10^ emphasizes that RV EF under stress is an important surrogate parameter for the contractile reserve and can identify occult RV‐PA uncoupling. When the reserve is limited, acute RV dilatation and a relevant decrease in EF occur.^10^ The SV/ESV ratio relates mathematically to EF: SV/ESV = EF/(1−EF).^44^ Higher ratios indicate stronger systolic performance, while lower values reflect increased ESV and heterometric adaptation. Cardiac magnetic resonance defines maladaptation thresholds (RV EF <35%, SV/ESV <0.54) and shows that in severe PAH submaximal exercise fails to raise RV EF or the coupling ratio, revealing limited contractile reserve.^45^, ^46^ SV/ESV was validated as a sensitive non‐invasive marker in severe PAH,^4^ with exercise studies in chronic thromboembolic PH linking reduced ratios to poorer outcomes.^47^

Our results add to this by showing that the course of ΔEes/Ea and the increase of Ees in particular are strongly associated with the clinical outcome. Our results are remarkably consistent with the observations of Grünig *et al*.,^9^ who were able to show that a large increase in sPAP during exercise indicates preserved RV contractility and is associated with a more favourable clinical outcome. Our data, which also associate a high ΔsPAP with a favourable (homeometric) RV adaptation, support this concept and underline the importance of a differentiated consideration of pressure and volume parameters under exercise conditions.

The distinction between the different types of RV adaptation is clinically important, as unfavourable RV adaptation may require closer monitoring or more intensive therapy. Since resting examinations can mask differences, stress tests that measure RV response (e.g. volume increase under stress) could improve patient classification. If PV catheterization is not available outside specialized centres, practical methods such as stress magnetic resonance imaging or stress 3D echocardiography, in which a ΔsPAP >30 mmHg can serve as a substitute for preserved contractile reserve, offer alternative approaches.^9^ Despite technical and patient‐specific challenges, they allow a more accurate assessment of RV function than isolated haemodynamic examinations.

### Limitations

The study has several limitations. First, our sample size was limited. However, PV catheter characterization in patients with PH is rare, and the size of our cohort is comparable to or larger than the cohort sizes of previously published PV catheter studies, particularly considering the technical complexity, patient selection criteria, and the invasive nature of the method which limits broad applicability in routine clinical settings. Second, we did not include healthy individuals or patients from PH groups 2 and 3. Third, there were missing spiroergometer data for some of the patients. These factors introduce some heterogeneity and bias so further studies are needed to validate our findings in a range of patients from diverse groups including those from PH groups 2 and 3.

## Conclusions

Our study reveals two distinct RV adaptation patterns under stress in PH: a homeometric adaptation (high contractile reserve and limited volume increase) and a heterometric dilation strategy (low contractile reserve and greater volume increase). This distinction has significant prognostic relevance. While the classic mPAP/CO slope inadequately reflects this distinction in advanced PH, ΔsPAP and the cardiac index under peak exercise allow the identification of the RV adaptation types and thus enable a differentiated prognosis with regard to RV functional status.

## Supporting information


**Appendix S1.** Supporting Information.
