# Supplementary material for: Exercise haemodynamics in pulmonary hypertension – a prospective pressure–volume loop study on right ventricular adaptation and prognosis
Source: Eur J Heart Fail. 2025 Aug 7;27(12):3180–92. doi: 10.1002/ejhf.3802 (PMC12803536; doi:10.1002/ejhf.3802)

**SUPPORTING INFORMATION**

**Exercise haemodynamics in pulmonary hypertension – a prospective pressure-volume loop study on right ventricular adaptation and prognosis**

Bruno R. Thal, Zvonimir A. Rako, Nils C. Kremer, Athiththan Yogeswaran, Patrick Janetzko, Selin Yildiz, Stephan Rosenkranz, Hossein Ardeschir Ghofrani, Werner Seeger, Friedrich Grimminger and Khodr Tello


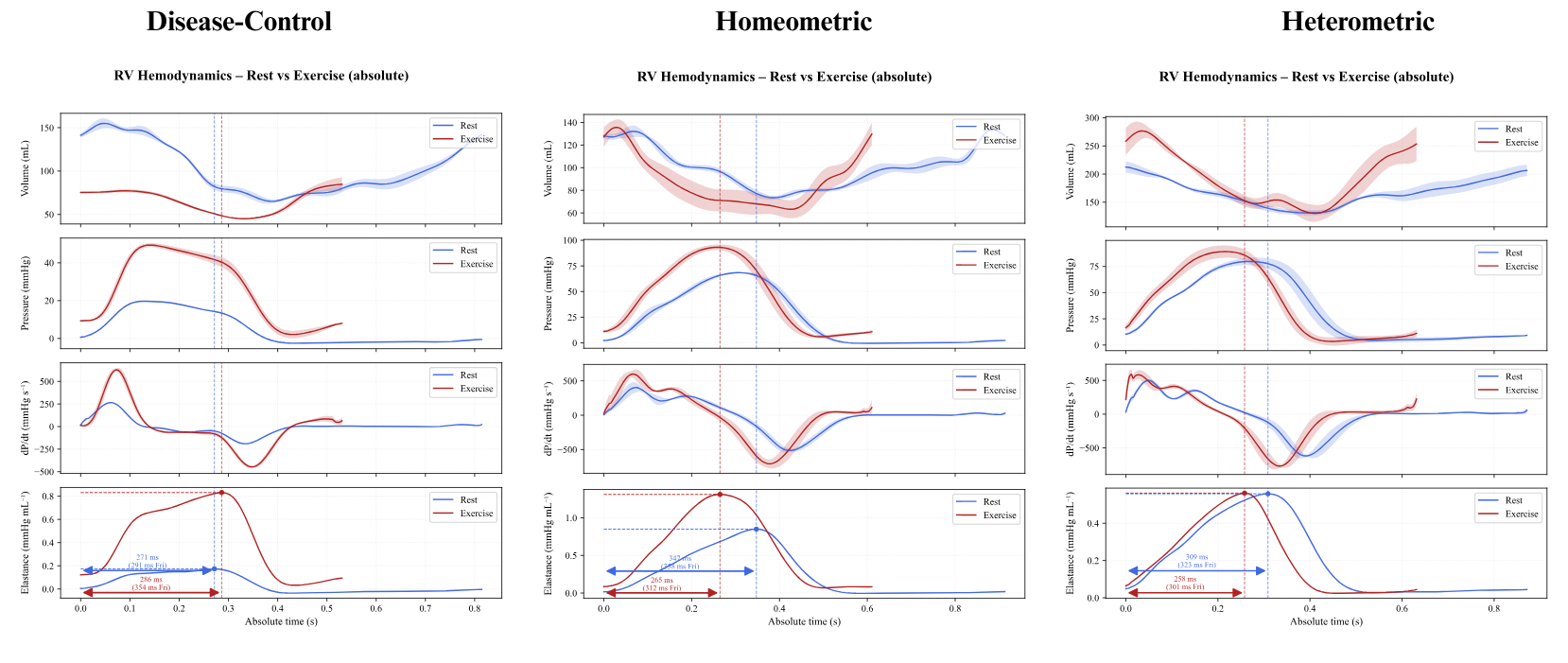


**Figure S1** –Representative RV haemodynamics at rest (blue) and during exercise (red) in disease control, homeometric, and heterometric groups. Shaded areas indicate 95% confidence intervals. Dotted lines mark peak elastance, and arrows show Fridericia-corrected systolic ejection time. Fri, Fridericia; RV, right ventricular.


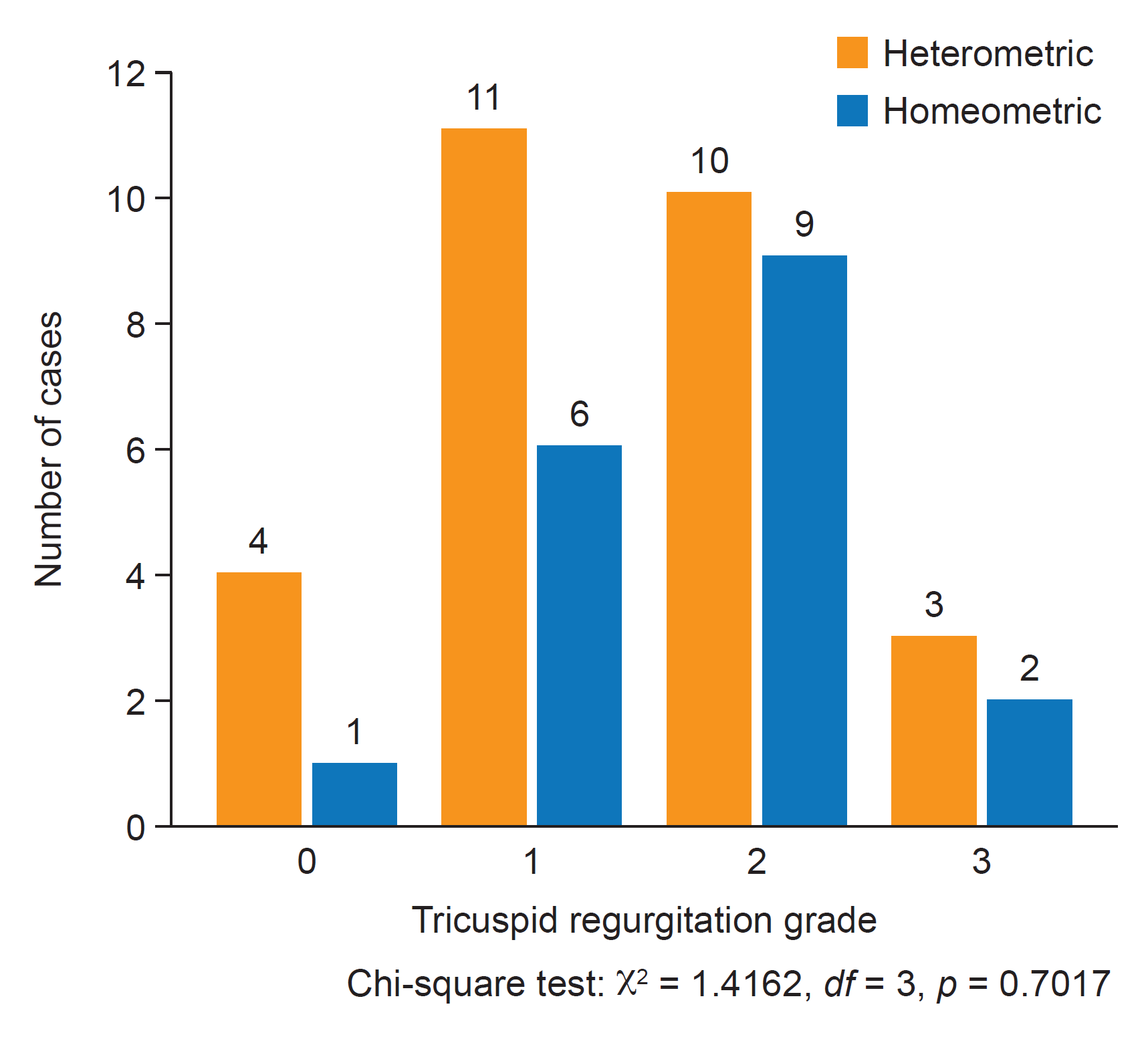


**Figure S2 –** Distribution of tricuspid regurgitation grades in patients with pulmonary hypertension and heterometric (*n* = 28) or homeometric (*n* = 18) right ventricular adaptation to exercise. The number of cases is indicated above each bar. A chi-square test indicates no statistically significant difference between the groups.


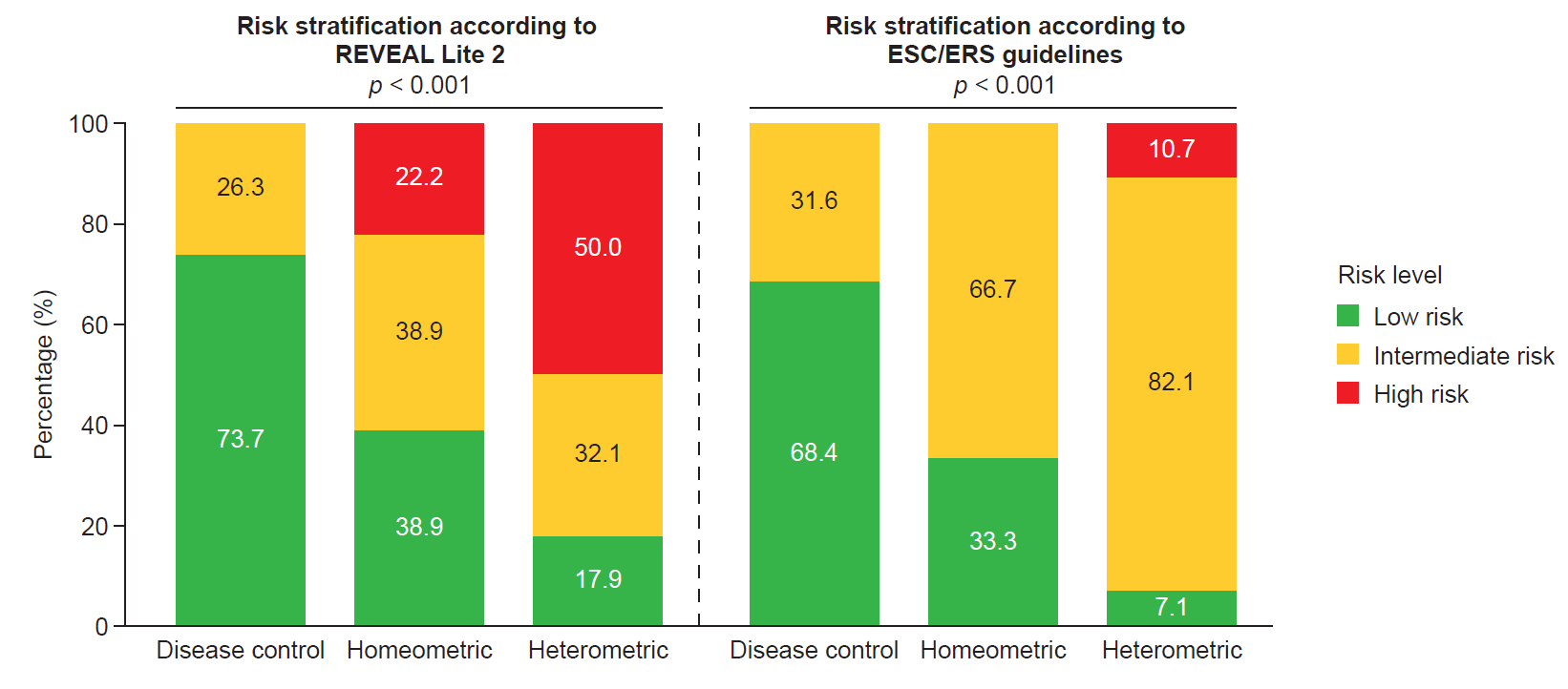


**Figure S3 –** Risk stratification according to the REVEAL Lite 2 and ESC/ERS guideline-based risk scores in disease controls (patients with invasive exclusion of pulmonary hypertension; *n* = 19) and patients with pulmonary hypertension and heterometric (*n* = 28) or homeometric (*n* = 18) right ventricular adaptation to exercise. Heterometric adaptation was associated with the highest overall risk. ESC/ERS, European Society of Cardiology/European Respiratory Society.


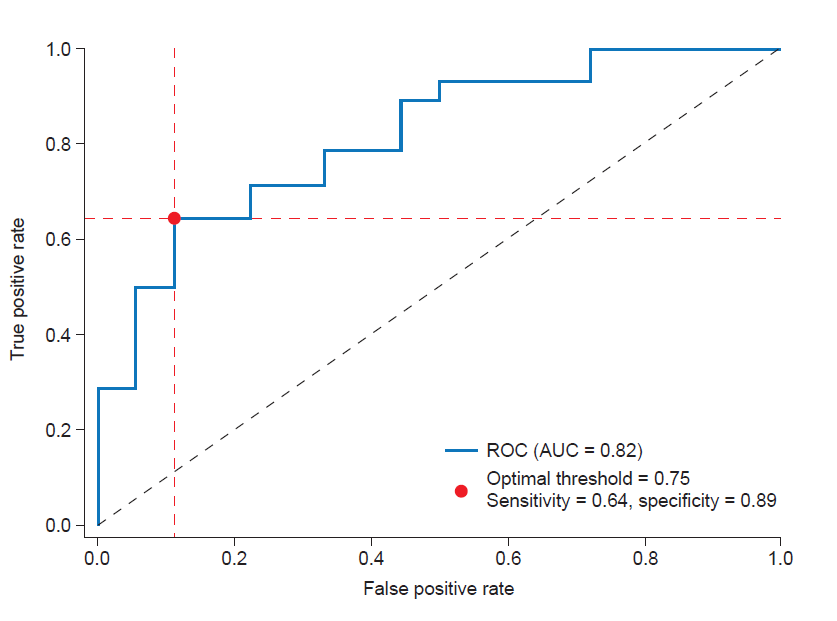


**Figure S4 –** ROC analysis of the ability of the final multivariable logistic regression model to distinguish right ventricular homeometric and heterometric adaptation. The final logistic regression model, which included peak cardiac index and the change in systolic pulmonary arterial pressure from rest to peak exercise, achieved an AUC of 0.82. At an optimal threshold of 0.75 (per Youden's index), the model yielded a sensitivity of 64% and a specificity of 89%. AUC, area under the curve; ROC, receiver operating characteristic.

**Table S1 – LV parameters at rest**

| **LV parameters at rest** | **Total** | **Disease control** | **Homeometric group** | **Heterometric group** | ***p*^a^** |
| --- | --- | --- | --- | --- | --- |
| LA ESA, cm² | 17.8 ± 4.6 | 17.1 ± 3.6 | 18.7 ± 4.3 | 17.8 ± 5.4 | 0.546ᵇ |
| E max, cm/s | 63.8 [53.6, 85.3] | 65.1 [54.4, 87.8] | 63.2 [57.9, 71.8] | 64.0 [48.6, 88.1] | 0.991ᶜ |
| Med e’ max, cm/s | 6.5 ± 1.6 | 7.1 ± 1.5 | 6.5 ± 1.3 | 6.1 ± 1.7 | 0.385ᵇ |
| E/A (*n*^d^) | 0.9 [0.7, 1.1] (*n* = 2) | 0.9 [0.7, 1.2] | 0.8 [0.7, 1.0] (*n*= 1) | 0.9 [0.7, 1.1] (*n*= 1) | 0.791ᶜ |
| E/lat e’ (*n*^d^) | 7.3 [5.4, 9.5] (*n*= 17) | 9.2 [7.6, 10.1] (*n*= 7) | 5.6 [5.2, 6.7] (*n*= 3) | 7.6 [6.2, 9.4] (*n*= 7) | 0.124ᶜ |
| LV GLS, % | −21.6 ± 3.2 | −23.1 ± 2.5 | −22.0 ± 3.5 | −20.3 ± 3.1 | 0.104ᵇ |
| 3D LA EF, % (*n*^d^) | 53.3 ± 14.2 (*n*= 4) | 60.3 ± 12.6 (*n*= 1) | 54.7 ± 14.2 (*n*= 2) | 47.8 ± 13.5 (*n*= 1) | 0.121ᵇ |
| 3D LAVI, ml/m² (*n*^d^) | 33.1 [26.0, 38.0] (*n*= 4) | 34.0 [22.2, 35.8] (*n*= 1) | 34.5 [27.8, 39.2] (*n*= 2) | 33.0 [27.0, 39.0] (*n*= 1) | 0.880ᶜ |
| 3D LV EDV, ml (*n*^d^) | 122.1 ± 32.7 (*n*= 2) | 131.8 ± 31.0 | 124.6 ± 30.2 (*n*= 1) | 113.7 ± 34.3 (*n*= 1) | 0.288ᵇ |
| 3D LV ESV, ml (*n*^d^) | 48.0 [35.0, 58.0] (*n*= 2) | 54.0 [39.5, 58.0] | 50.0 [41.0, 56.0] (*n*= 1) | 42.0 [34.0, 55.0] (*n*= 1) | 0.334ᶜ |
| 3D LV SV, ml (*n*^d^) | 71.6 ± 19.4 (*n*= 2) | 79.2 ± 15.3 | 69.8 ± 22.0 (*n*= 1) | 67.3 ± 19.3 (*n*= 1) | 0.685ᵇ |
| 3D LV EF, % (*n*^d^) | 60.1 ± 5.8 (*n*= 2) | 60.7 ± 4.8 | 60.1 ± 5.4 (*n*= 1) | 59.6 ± 6.7 (*n*= 1) | 0.811ᵇ |

Values represent mean ± standard deviation or median [first quartile, third quartile], unless otherwise specified.

E, early mitral inflow velocity; e’, mitral annular early diastolic tissue velocity; E/A, ratio of early mitral inflow velocity to late diastolic transmitral flow velocity; EDV, end-diastolic volume; EF, ejection fraction; ESA, end-systolic area; ESV, end-systolic volume; GLS, global longitudinal strain; LA, left atrial; lat, lateral annulus; LAVI, left atrial volume index; LV, left ventricular; max, maximum; med, medial annulus; SV, stroke volume

^a^Homeometric versus heterometric group

^b^t-test

^c^Mann–Whitney U test

^d^Number of participants with missing data

**Table S2 – Haemodynamics and RV function at rest and peak exercise**

| **Parameter** | **Rest** | **Peak exercise** | ***p^a^*** |
| --- | --- | --- | --- |
| Disease control (*n* = 19) |  |  |  |
| EDP, mmHg | 3.8 ± 3.2 | 6.5 ± 6.7 | 0.012^b^ |
| ESP, mmHg | 20.6 ± 7.5 | 35.3 ± 12.1 | <0.001^b^ |
| EDV, ml | 105.0 ± 29.4 | 105.6 ± 27.7 | 0.91^b^ |
| ESV, ml | 43.0 [40.0, 54.0] | 55.0 [43.0, 58.0] | 0.236^c^ |
| SV, ml | 69.6 ± 16.5 | 77.5 ± 29.4 | 0.179^b^ |
| Eed, mmHg/ml | 0.2 ± 0.1 | 0.2 [0.1, 0.3] | 0.045^c^ |
| Ees, mmHg/ml | 0.4 [0.3, 0.7] | 0.8 ± 0.4 | 0.001^c^ |
| Ea, mmHg/ml | 0.3 [0.2, 0.4] | 0.5 ± 0.3 | <0.001^c^ |
| Ees/Ea | 1.6 ± 0.5 | 1.7 ± 0.6 | 0.471^b^ |
| 3D RV EF, % (*n* = 2 with missing data) | 51.1 ± 4.7 | 48.6 ± 7.1 | 0.179^b^ |
| mPAP, mmHg | 16.5 ± 2.7 | 27.1 ± 7.0 | <0.001^b^ |
| Cardiac index, l/min/m^2^ | 3.0 ± 0.7 | 5.0 ± 1.8 | <0.001^b^ |
| Homeometric group (*n* = 18) |  |  |  |
| EDP, mmHg | 5.5 [3.2, 7.5] | 13.9 ± 7.3 | <0.001^c^ |
| ESP, mmHg | 56.7 ± 22.7 | 91.2 ± 24.3 | <0.001^b^ |
| EDV, ml | 143.9 ± 41.8 | 147.8 ± 41.7 | 0.374^b^ |
| ESV, ml | 83.1 ± 28.8 | 83.3 ± 32.6 | 0.957^b^ |
| SV, ml | 78.8 ± 25.3 | 99.9 ± 36.2 | 0.001^b^ |
| Eed, mmHg/ml | 0.2 ± 0.1 | 0.3 [0.2, 0.3] | 0.054^c^ |
| Ees, mmHg/ml | 0.8 ± 0.3 | 1.3 ± 0.4 | <0.001^b^ |
| Ea, mmHg/ml | 0.7 [0.4, 1.1] | 1.0 [0.7, 1.2] | <0.001^c^ |
| Ees/Ea | 1.1 ± 0.3 | 1.4 ± 0.4 | <0.001^b^ |
| 3D RV EF, % (*n* = 3 with missing data) | 43.8 ± 9.2 | 42.6 ± 10.4 | 0.365^b^ |
| mPAP, mmHg | 38.9 ± 11.9 | 61.9 ± 18.1 | <0.001^b^ |
| Cardiac index, l/min/m^2^ | 2.7 [2.5, 3.0] | 4.1 ± 0.8 | 0.001^c^ |
| Table continues on next page. | | | |
| Heterometric group (*n* = 28) |  |  |  |
| EDP, mmHg | 9.4 ± 5.8 | 17.7 ± 9.3 | <0.001^b^ |
| ESP, mmHg | 58.4 ± 20.9 | 81.8 ± 19.7 | <0.001^b^ |
| EDV, ml | 143.2 ± 48.3 | 164.3 ± 46.3 | <0.001^b^ |
| ESV, ml | 88.4 ± 35.2 | 109.4 ± 42.4 | <0.001^b^ |
| SV, ml | 80.0 ± 20.6 | 76.1 ± 25.7 | 0.494^b^ |
| Eed, mmHg/ml | 0.2 [0.1, 0.3] | 0.4 ± 0.2 | <0.001^c^ |
| Ees, mmHg/ml | 0.8 [0.6, 1.0] | 0.9 ± 0.3 | 0.12^c^ |
| Ea, mmHg/ml | 0.6 [0.5, 0.9] | 1.0 [0.9, 1.3] | <0.001^c^ |
| Ees/Ea | 1.1 ± 0.3 | 0.9 ± 0.4 | <0.001^b^ |
| 3D RV EF, % (*n* = 3 with missing data) | 40.5 ± 9.5 | 36.2 ± 9.8 | 0.003^b^ |
| mPAP, mmHg | 40.2 ± 12.1 | 58.1 ± 16.1 | <0.001^b^ |
| Cardiac index, l/min/m^2^ | 2.5 ± 0.6 | 3.3 ± 1.0 | <0.001^b^ |

Values represent mean ± standard deviation or median [first quartile, third quartile], unless otherwise specified.

3D, three-dimensional; Ea, arterial elastance; EDP, end-diastolic pressure; EDV, end-diastolic volume; Eed, end-diastolic elastance; Ees, end-systolic elastance; EF, ejection fraction; ESP, end-systolic pressure; ESV, end-systolic volume; mPAP, mean pulmonary arterial pressure; RV, right ventricular; SV, stroke volume

^a^Rest versus peak exercise

^b^Paired t-test

^c^Wilcoxon test

**Table S3 – Additional exercise haemodynamic parameters**

| **Exercise haemodynamics** | **Total** | **Disease control** | **Homeometric group** | **Heterometric group** | ***p^a^*** |
| --- | --- | --- | --- | --- | --- |
| RHC – peak |  |  |  |  |  |
| dPAP, mmHg | 29.0 [18.0, 39.0] | 16.0 [14.0, 18.0] | 35.0 [29.0, 43.2] | 34.5 [26.0, 46.0] | 0.839ᶜ |
| PAPi | 4.3 [3.1, 6.0] | 4.4 [3.2, 5.5] | 5.7 [4.5, 7.0] | 3.5 [2.9, 4.6] | 0.007ᶜ |
| TPR, WU | 6.7 [4.0, 10.2] | 2.6 [1.8, 4.0] | 7.9 [6.2, 9.7] | 8.9 [6.6, 14.6] | 0.295ᶜ |
| TPG, mmHg | 36.8 ± 20.4 | 15.5 ± 5.3 | 49.5 ± 19.7 | 43.0 ± 16.0 | 0.229ᵇ |
| RHC – response to exercise |  |  |  |  |  |
| Δ dPAP, mmHg | 7.0 [4.0, 13.0] | 5.0 [2.0, 7.5] | 12.0 [6.0, 18.8] | 8.5 [5.0, 13.2] | 0.379ᶜ |
| Δ mPAP, mmHg | 16.0 [9.0, 24.0] | 9.0 [6.5, 14.0] | 23.5 [16.8, 30.0] | 16.5 [11.0, 24.0] | 0.054ᶜ |
| Δ PVR, dyn·s/cm^5^ | 18.1 [−41.6, 66.9] | −12.3 [−19.1, 21.6] | 30.1 [8.1, 80.1] | 26.7 [−67.3, 86.0] | 0.581ᶜ |
| Δ PAPi (*n*^d^) | −0.3 [−2.1, 1.2] (*n*= 2) | 1.1 [−0.2, 2.0] | −1.3 [−4.5, 0.4] (*n*= 2) | −0.7 [−1.9, 0.0] | 0.457ᶜ |
| Δ Heart rate, beats/min | 29.0 [21.0, 42.0] | 35.0 [25.5, 48.5] | 30.0 [21.5, 49.0] | 24.5 [17.0, 33.2] | 0.146ᶜ |
| Δ RAP, mmHg | 4.8 ± 5.5 | 0.5 ± 4.6 | 5.8 ± 4.7 | 7.1 ± 4.9 | 0.374ᵇ |
| Δ CO, l/min | 3.1 ± 2.3 | 4.8 ± 3.0 | 2.8 ± 1.6 | 2.1 ± 1.2 | 0.104ᵇ |
| Δ Cardiac index, l/min/m² | 1.3 ± 1.2 | 2.1 ± 1.5 | 1.1 ± 1.0 | 0.8 ± 0.8 | 0.288ᵇ |
| Δ RAP/CO, mmHg·min/l | 0.4 ± 1.1 | −0.2 ± 0.8 | 0.5 ± 0.8 | 0.8 ± 1.2 | 0.478ᵇ |
| Δ TPR, WU | 0.2 ± 1.5 | −0.2 ± 1.1 | 0.5 ± 1.1 | 0.3 ± 2.0 | 0.645ᵇ |
| Δ TPG, mmHg | 13.2 ± 9.0 | 6.8 ± 5.1 | 18.9 ± 10.1 | 13.8 ± 7.8 | 0.062ᵇ |

Values represent mean ± standard deviation or median [first quartile, third quartile], unless otherwise specified. Δ (change during exercise) indicates the difference between rest and peak exercise measurements for the respective parameter.

CO, cardiac output (assessed by thermodilution); dPAP, diastolic pulmonary arterial pressure; mPAP, mean pulmonary arterial pressure; PAPi, pulmonary arterial pulsatility index; RAP, right atrial pressure; RHC, right heart catheterization; sPAP, systolic pulmonary arterial pressure; TPG, transpulmonary gradient; TPR, total pulmonary resistance; WU, Wood Units

^a^Homeometric versus heterometric group

^b^t-test

^c^Mann–Whitney U test

^d^Number of participants with missing data

**Table S4 – Systolic ejection time at rest and during exercise across groups**


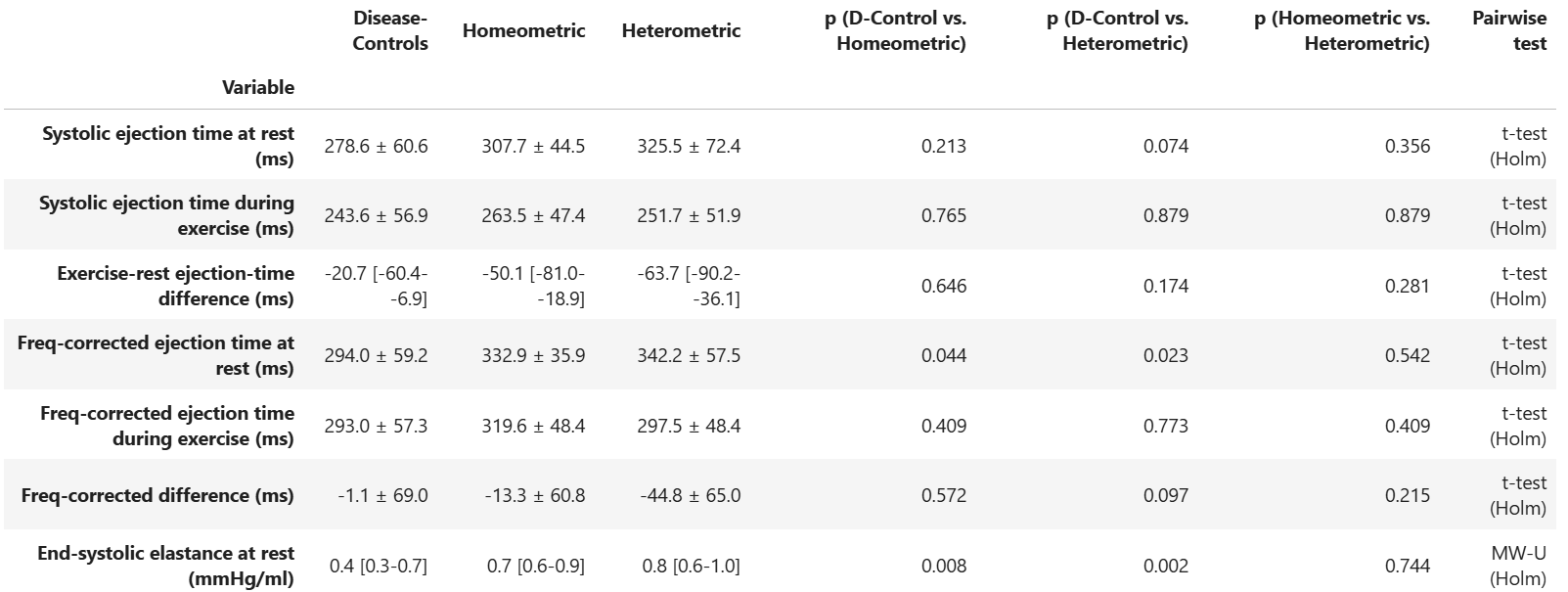

Supplement: Supplementary file 1 — Appendix S1. Supporting Information. [file EJHF-27-3180-s001.docx]
